# Relationship between Breast Cancer Surgical Treatment and Psychiatric Symptomatology: Which Sociodemographic and Clinical Factors Could Influence It? A Preliminary Study

**DOI:** 10.3390/bs12010009

**Published:** 2022-01-06

**Authors:** Ilaria Baldelli, Matteo Gari, Andrea Aguglia, Andrea Amerio, Valeria Berrino, Gregorio Santori, Daniele Friedman, Gianluca Serafini, Mario Amore, Edoardo Raposio

**Affiliations:** 1 Department of Surgical Sciences and Integrated Diagnostics (DISC), University of Genoa, 16132 Genoa, Italy; ilaria.baldelli@unige.it (I.B.); vale_berrino@hotmail.it (V.B.); gregorio.santori@gmail.com (G.S.); friedman@unige.it (D.F.); edoardo.raposio@unige.it (E.R.); 2 IRCCS Ospedale Policlinico San Martino, 16132 Genoa, Italy; mattegari@me.com (M.G.); andrea.amerio@unige.it (A.A.); gianluca.serafini@unige.it (G.S.); mario.amore@unige.it (M.A.); 3 Department of Neuroscience, Rehabilitation, Ophthalmology, Genetics, Maternal and Child Health (DINOGMI), Section of Psychiatry, University of Genoa, 16132 Genoa, Italy

**Keywords:** breast cancer, depression, stress, surgery, anxiety, hopelessness

## Abstract

This study aimed to investigate psychiatric symptomatology in a sample of patients affected by breast cancer undergoing surgery, evaluating the potential mediators on perceived stress levels, depression and hopelessness. The study was conducted on eighty-five patients with breast cancer, admitted consecutively to the Breast Unit of the IRCCS Ospedale Policlinico San Martino, between May 2018 and December 2019. Sociodemographic (age of diagnosis, gender, marital and occupational status, educational level, having children) and clinical (type and side of surgery, previous breast surgery, neoadjuvant chemotherapy and axillary dissection) characteristics were investigated through a semi-structured interview. The following rating scales were administered: Beck Depression Inventory, Beck Hopelessness Scale, and Perceived Stress Scale. Our findings indicate that the presence of children and of a partner was associated with a lower total score on the clinical dimensions evaluated. Furthermore, we found demolitive surgery to be a mediator between perceived stress and hopelessness, while history of previous breast surgery was found to be a mediator between demolitive surgery and perceived stress. In conclusion, patients affected by breast cancer undergoing more complex and demolitive surgery or with history of previous breast surgery should be mostly monitored from a psychological and psychiatric point of view from the beginning of treatments to evaluate the first manifestations of psychiatric symptomatology.

## 1. Introduction

In 2020, breast cancer was the most frequently diagnosed cancer in the Italian population, with an increased incidence year by year, especially in the non-screening age groups and in the north-central areas. It is due to both the increase of screening exams and programs of prevention and a more specific awareness of lifestyle-related risk factors. Breast cancer is characterized by an overall good prognosis, with 53,000 new diagnoses in 2019 alone and 12,000 related deaths [1].

In recent years, better early detection and diagnosis have led to a more effective treatment of patients with breast cancer and, therefore, women affected live longer [2]. Considering the fundamental role that women play in modern society as workers, partners, wives and mothers, the different approach offered during treatment and after healing is a crucial issue, having a significant impact on mental health, quality of life (QoL) and global functioning. Therefore, the management of a patient with breast cancer needs to achieve different goals [3,4,5]. Its evaluation is inextricably related to the effects of surgical procedures and the consequent implications on perception of the body. Cancer diagnosis inevitably interferes with all aspects of patients’ daily life, provoking wide and different inter-individual responses [6]. For example, breast cancer related worries, such as the fear of feeling less attractive with an altered body image for surgical interventions, are added to the common aspects of all patients affected by cancer, such as pain, fear of recurrence and fatigue [7]. The different surgical approaches, although more respectful and of multidisciplinary competence, could still have a negative psychological and emotional impact [5], including perception of one’s body image [8]. 

Body image perception seems to be better following breast conservative than demolitive surgery, independently with or without immediate reconstructive surgery. However, a panel of experts in breast cancer disagree, affirming that body image perception may be probably influenced by unknown factors other than surgical techniques and cosmetic outcomes [9].

Among patients with breast cancer, psychiatric symptoms, such as somatic and anxiety, depression and maladaptive coping strategies, occur in between 10% and 40% of patients [6,10,11,12], evaluated between the diagnosis and treatment course until survivorship or illness recurrences [13]. However, the highest level of distress is experienced between preliminary diagnosis and surgery, because of the concern for definitive diagnosis and necessary postoperative treatment [14]. 

The uncertainty of the future, characteristic of those diagnosed with cancer, can frequently lead patients to experience hopelessness [15], which is as a particular emotional state that makes individuals unable to solve problems and activate the energy to fulfil their goals. A 24-year longitudinal study showed how hopelessness was also associated with less participation in breast cancer screenings, thus resulting in less breast cancer incidents reported [16]. This could play an important role in patients’ compliance with the disease and treatment related to breast cancer, where hopelessness may be a significant predictor of suicidal thoughts and behaviors [17,18] and depression a prognostic factor for breast cancer mortality [19]. 

Therefore, this study aimed to investigate if the presence of particular demographic, social and medical factors, at the time of surgical treatment, could be related to the presence of psychiatric symptoms, such as perceived stress and increased levels of hopelessness. The identification of subjective characteristics, related to the presence of high perioperative stress, could be useful in planning standard interventions, aimed at support during postoperative adjuvant treatments.

## 2. Materials and Methods

### 2.1. Participants

The study was conducted on a sample of 85 patients with a primary diagnosis of breast cancer, admitted consecutively to the Breast Surgery Unit of the IRCCS Ospedale Policlinico San Martino (Genoa, Italy), between May 2018 and December 2019. 

The following inclusion criteria were considered: age ≥18 years, diagnosis of breast cancer in all stages of the disease, awareness of the diagnosis and willingness to sign a written informed consent. The exclusion criteria were: history of oncological disease of a different nature, presence of current cognitive or psychiatric disorders, according to diagnostic and statistical manual for mental disorders—fifth edition (a psychiatrist with at least ten year of experience visited patients before enrolment), physical limitations or severity of the disease to be unable to complete evaluation scales and participate to the study. 

### 2.2. Assessment 

A semi-structured questionnaire was used to assess patients’ basic sociodemographic characteristics, including gender, age, marital and occupational status, educational level, and having children. Clinical information included history of previous breast surgery for cancer, neoadjuvant chemotherapy, type of surgery (conservative vs. demolitive), side of surgery (unilateral vs. bilateral), and axillary dissection.

In addition, the following rating scales were administered:

‑ The Beck Depression Inventory (BDI) [20] is a 21-item, self-reported rating inventory, for measuring characteristic attitudes and symptoms of depression in general and psychiatric populations. The total score ranges from 0 to 63: 0–13 is considered a minimal range, 14–19 mild, 20–28 moderate and 29–63 indicates severe depressive symptomatology.
‑ The Perceived Stress Scale (PSS) [21] is the most widely used psychological instrument for measuring the perception of stress. It is a measure of the degree to which situations in one’s life are appraised as stressful. Items are designed to tap into how unpredictable, uncontrollable, and overloaded respondents find their lives. The questions refer to feelings and thoughts during the last month.
‑ The Beck Hopelessness Scale (BHS) [22] is used to assess the severity of hopelessness symptoms. The BHS consists of 20 true-or-false items. BHS scores are categorized into: normal (0–3), mild (4–8), moderate (9–14) and severe hopelessness (15–20). Research supports a significant association between BHS scores, depression, suicidal intent and current suicidal ideation. A BHS cut-off score of 9 or higher is considered for individuals at increased suicidal risk [23].

All participants received a detailed explanation of the study design and a written informed consent was obtained from all respondents, according to the guidelines provided in the current version of the Declaration of Helsinki. The study design was approved by the local Ethical Review Board (098/2018).

### 2.3. Statistical Analysis 

The continuous variables were represented as mean and standard deviation (SD), while categorical variables were represented as frequency and percentage, considering sociodemographic and clinical characteristics. The Shapiro-Wilk test was preliminarily used to assess the normal distribution of continuous variables. Based on the results of the Shapiro-Wilk test, the non-parametric Mann–Whitney U-test was used to compare continuous variables between groups. Correlation analysis was performed by using the Spearman’s rank correlation. 

Statistical significance was set with *p* value < 0.05 (two-tailed). We carried out the statistical analyses using the Statistical Package for Social Sciences (IBM Corp., Armonk, NY, USA) for Windows 25.0 and the R statistical environment (version 4.0.3, R Foundation for Statistical Computing, Vienna, Austria).

Finally, two mediation analyses were carried out: the first between perceived stress (independent variable) and hopelessness feelings (dependent variable) via demolitive surgery (mediator). The second one between demolitive surgery (independent variable) and perceived stress (dependent variable) via history of previous breast surgery for cancer (mediator). Linear regression analyses were conducted, and the Sobel test was used to examine and confirm the simple mediation analysis [24].

## 3. Results

Eighty-five patients were enrolled and the current age was 59.93 ± 14.03. Sociodemographic and clinical characteristics are shown in Table 1. Almost two-thirds of patients (63.5%) received breast-conserving surgery, in which only a portion of the breast was removed. When mastectomy was performed, 71% of patients (*n* = 22) received breast reconstruction. In the majority of patients (95.3%), surgery was unilateral. Only 16.5% of patients underwent removal of axillary lymph nodes due to their involvement.

In patients with a stable relationship, an association with lower total scores of BDI (3.49 ± 5.00 vs. 6.86 ± 7.88, *p* = 0.022) and BHS (2.13 ± 2.08 vs. 5.04 ± 5.53, *p* = 0.050) was identified compared to single patients.

Regarding to occupational status, being employed significantly correlated only with the age of the patient (*p* < 0.001) (Table 2).

A higher age at diagnosis (62.82 ± 13.27 vs.52.13 ± 13.25, *p* = 0.001) was statistically associated with having children and with lower total scores of PSS (9.26 ± 8.12 vs. 14.44 ± 8.87, *p* = 0.019), compared to not having children.

Lower total scores of PSS (9.28 ± 7.97 vs. 13.20 ± 9.24, *p* = 0.048) was associated to a conservative type of surgery. Moreover, bilateral surgery was associated with higher total scores of BHS (9.00 ± 4.97 vs. 2.88 ± 3.54, *p* = 0.007) and lower age at diagnosis (45.75 ± 3.30 vs. 60.63 ± 13.99, *p* = 0.016). Undergoing axillary surgery did not show association with any variable, however patients who had received it showed, in particular, higher total scores of BHS (5.00 ± 5.36 vs. 2.80 ± 3.36, *p* = 0.050).

**Figure 1:**
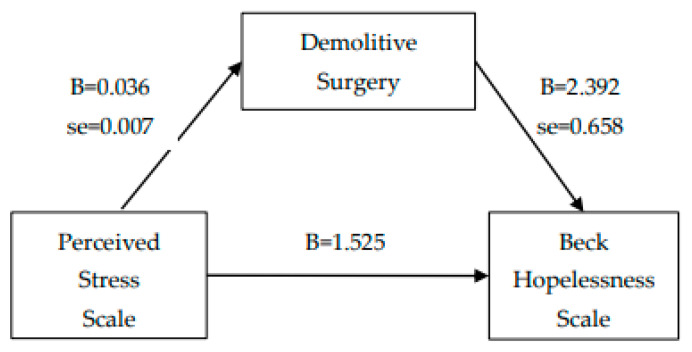
Mediation analysis_1 (Sobel test = 2.969, SE = 0.029, *p* = 0.002).

From simple linear regression, perceived stress was a statistically significant predictor of hopelessness feelings. Furthermore, perceived stress was also a significant predictor of the mediating variable (demolitive surgery). When the mediator, demolitive surgery, was entered in the regression analysis, perceived stress was no longer a significant predictor of hopelessness feelings. Finally, the Sobel test confirmed demolitive surgery as a potential mediator of the association between perceived stress and hopelessness feelings (see Figure 1).

**Figure 2:**
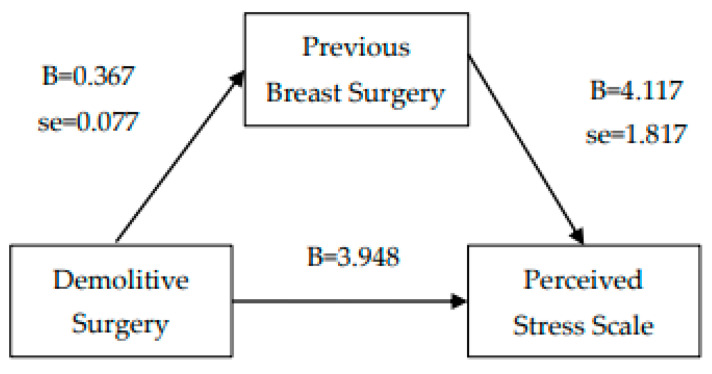
Mediation analysis_2 (Sobel test = 2.046, SE = 0.579, *p* = 0.041).

From simple linear regression, demolitive surgery was a statistically significant predictor of perceived stress. Furthermore, demolitive surgery was also a significant predictor of the mediating variable (previous breast surgery). When the mediator, previous breast surgery, was entered in the regression analysis, demolitive surgery was no longer a significant predictor of perceived stress. Finally, the Sobel test confirmed previous breast surgery as a potential mediator of the association between demolitive surgery and perceived stress (see Figure 2).

## 4. Discussion

The purpose of this study was to evaluate which sociodemographic and clinical factors had an impact on depression, hopelessness feelings and perceived stress in patients with a primary diagnosis of breast cancer in the immediate postoperative period.

We found that, regardless of the aesthetic result (assessable later), undergoing conservative surgery caused less stress in patients, probably because the idea of preserving most of the breast has less impact on breast-specific concerns, such as femininity and attractiveness. The extension of the screening campaigns allowed earlier diagnosis of the disease [25], opening the way to more conservative surgery, which is less prone to leading to the development of depression symptoms, compared to demolitive surgery [26]. However, postoperative QoL seems to be influenced by several unknown factors other than surgical techniques and clinical conditions [9]. For example, even if mastectomy has evolved into an increasingly less destructive treatment, in most cases with the preservation of the entire skin envelope including the areola-nipple complex, the postoperative QoL is still affected. The complete gland removal and the need for reconstruction affects the length of the postoperative hospital stay and pain, and the need for assistance during home recovery, such as managing of dressings and surgical drains or daily activities that require the use of the arms, can be significant for QoL.

Depression, hopelessness and perceived stress are frequently following the diagnosis of breast cancer [27], especially if lymph nodes were examined, because their positivity could predict an extremely demolitive intervention. Our study seems to confirm this trend, in particular with regard to hopelessness, that was detected in patients who had undergone axillary surgery. Similarly, probably due to the greater complexity of the surgical intervention, bilateral disease was associated with higher scores of BHS.

Protective factors for the development of psychopathological symptoms in patients with breast factors are studied in the literature. As shown in previous studies [28,29], the presence of a person to rely on and with whom to share the care’ path reduces stressful factors for the patient, in some cases precluding a reduction in depressive symptoms and an improvement in terms of sleep disturbances and management of stress. Yang et al. have also shown how the presence of distress in the family could lead to a lengthening of recovery times [30]. In our study, having children was associated to a lower susceptibility to experience higher perceived stress levels and being part of a stable relationship to lower depressive mood and hopelessness feelings; these factors should not be underestimated, instead clinicians need to take the whole family and their support needs into account [31], assessing also the potential psychological needs of children and partners, living with these patients [32]. Data suggested that the family-oriented interventions, such as “getting well together”, appears to be beneficial to mothers’ and children’s QoL and psychological well-being [33].

Having children also influences sociodemographic factors, such as an older age at diagnosis, confirming the protective role of pregnancy on breast cancer, but also prognostic factors, such as less recourse to chemotherapy, aiming at a lower invasiveness of the cancer. Combining oncological rehabilitation and preventive child-centered interventions might be a feasible approach, supporting patients with breast cancer and their children with the consequent improvement of their emotional state. The psychological and relational dimensions represent an element of peculiar importance in oncology. In fact, surgeons should also consider the emotional and affective reactions of patients and their families on a daily basis, developing a particular sensitivity related to the perception of signs of discomfort and the limits inherent in the patient’s adaptation possibilities to the disease. Sometimes the need—which may be a legal requirement—to inform the patient can be difficult to reconcile with the desire of surgeons to encourage the patient; the constant collaboration with psychiatrists and psychologists, who have acquired specific experience in cancer communication, will allow surgeons to better address these issues. Psycho-oncology responds to the need for specific reflection on the psychic processes involved in adapting patients to disease and evaluating their everyday life. Therefore, it must provide useful tools for the organization of the training of all healthcare workers, involved in this particular field, and propose effective strategies in psychological support for patients from the beginning of their therapeutic path. It is a set of knowledge and skills in progressive evolution, on which the professional identity of the psycho-oncologist is based.

In view of these positive trends, currently at 87% after five years, an integrated breast unit should treat the “aftermath” by identifying and treating early predictive indicators of psychiatric symptomatology [34,35], such as depressive symptoms or hopelessness feelings, which could lead to shortened recovery times [36].

Therefore, it is necessary to find efficient ways of detecting those patients who are in risk for psychological distress [37,38]. In the already burdened mental state after the diagnosis of breast cancer, hopelessness feelings add to the difficulties due to both therapeutic methods, and a series of social and familial problems, that negatively affect this condition [39,40].

We found that 31.7% of our patients undergoing breast surgery reported mild to severe depressive symptoms; a percentage confirmed by data available in recent literature [11,41,42], with a higher rate within the first year following the diagnosis [43]. Depressive symptoms could be found even five years after the initial diagnosis in approximately 15% [44] of patients, negatively affecting their psychosocial and emotional functioning, social roles, perceived subjective pain and overall physical well-being [45,46,47], with decreased survival rates [48].

Several limitations of the study should be mentioned. First, a small sample recruited from only one university hospital limits our ability to generalize our findings. Second, information on the severity of disability resulting from surgery, interference with mobility and the presence of other medical comorbidities was not evaluated, limiting our ability to control these factors in a potential multivariate analysis. Finally, the use of self-administered scales could lead to an overestimation of stress, hopelessness and depressive levels.

## 5. Conclusions

More complex and demolitive surgery and a lack of support from close family members are common features among patients who tend to exhibit psychiatric symptoms early on in the course of breast cancer treatment. The integration into common clinical practice of self-administered scales and an adequate specific psychiatric assessment, on a case-by-case basis, could allow for an early identification of possible disease trajectories and lead to early interventions. A multidisciplinary approach could lead to an improvement of the management of these patients, defining personalized pharmacological and psychotherapeutic strategies, and reducing the negative consequences of breast cancer in vulnerable populations.

## Figures and Tables

**Table 1:** Sociodemographic and clinical characteristics of the sample.

	*N = 85*
Age at diagnosis (years), mean ± SD	59.93 ± 14.03
Occupational status, N (%)	
Employed	44 (51.8)
Not employed	41 (48.2)
Marital status, N (%)	
Couple	53 (62.4)
Single	28 (32.9)
Missing data	4 (4.7)
Having children, N (%)	
Yes	62 (72.9)
No	23 (27.1)
Type of surgery, N (%)	
Conservative	54 (63.5)
Demolitive	31 (36.5)
Side of surgery, N (%)	
Unilateral	81 (95.3)
Bilateral	4 (4.7)
Axillary dissection, N (%)	
Yes	14 (16.5)
No	71 (83.5)
Perceived Stress Scale, mean ± SD	10.68 ± 8.56
Beck Depression Inventory, mean ± SD	4.62 ± 6.23
Beck Hopelessness Scale, mean ± SD	3.17 ± 3.81

**Table 2:** Comparison of continuous variables between subgroups (non-parametric Mann–Whitney U-test).

	Age of Diagnosis (Years), Mean ± SD	*p*	PSS, Mean ± SD	*p*	BDI, Mean ± SD	*p*	BHS, Mean ± SD	*p*
Marital status, N (%)								
Single	62.00 ± 15.88	0.323	11.71 ± 9.40	0.688	6.86 ± 7.88	0.022	5.04 ± 5.53	0.05
Coupled	58.67 ± 13.47		10.65 ± 8.11		3.49 ± 5.00		2.13 ± 2.08	
Occupational status, N (%)								
Employed	52.34 ± 9.81	<0.001	10.30 ± 8.93	0.563	3.71 ± 5.61	0.114	3.50 ± 3.90	0.353
Not employed	68.07 ± 13.35		11.10 ± 8.31		5.61 ± 6.76		2.81 ± 3.73	
Having children, N (%)								
Yes	62.82 ± 13.27	0.001	9.26 ± 8.12	0.019	4.73 ± 6.15	0.689	2.94 ± 3.44	0.582
No	52.13 ± 13.25		14.44 ± 8.87		4.35 ± 6.56		3.78 ± 4.70	
Type of surgery, N (%)								
Conservative	60.63 ± 14.92	0.378	9.28 ± 7.97	0.048	4.70 ± 6.25	0.904	2.59 ± 3.16	0.162
Demolitive	58.71 ± 12.45		13.20 ± 9.24		4.48 ± 6.29		4.16 ± 4.63	
Side of surgery, N (%)								
Unilateral	60.63 ± 13.99	0.016	10.44 ± 8.50	0.219	4.56 ± 6.23	0.431	2.88 ± 3.54	0.007
Bilateral	45.75 ± 3.30		15.50 ± 10.41		6.00 ± 6.93		9.00 ± 4.97	
Neoadjuvant chemotherapy, N (%)								
Yes	54.00 ± 12.83	0.198	14.29 ± 8.04	0.237	6.14 ± 7.86	0.605	2.29 ± 3.50	0.237
No	60.46 ± 14.08		10.35 ± 8.62		4.49 ± 6.10		3.24 ± 3.85	
Axillary dissection, N (%)								
Yes	57.92 ± 12.64	0.561	14.30 ± 11.90	0.244	7.79 ± 8.58	0.099	5.00 ± 5.36	0.05
No	60.32 ± 14.33		10.01 ± 7.78		4.00 ± 5.52		2.80 ± 3.36	

PSS = Perceived Stress Scale; BDI = Beck Depression Inventory; BHS = Beck Hopelessness Scale.

## Data Availability

The data are not publicly available due to privacy/ethical restrictions.
